# Supplementary material for: Use of telemedicine in general practice in Europe since the COVID-19 pandemic: A scoping review of patient and practitioner perspectives
Source: PLOS Digit Health. 2024 Feb 14;3(2):e0000427. doi: 10.1371/journal.pdig.0000427 (PMC10866456; doi:10.1371/journal.pdig.0000427)
Supplement: Attachment — Submitted filename: Response to reviewers.docx [file pdig.0000427.s002.docx]

Dear Editor,

We would like to thank you and the reviewers for the valuable feedback of our manuscript ‘Use of telemedicine in general practice in Europe since the COVID-19 pandemic: a scoping review of patient and practitioner perspectives’ (PDIG-D-22-00363). We have revised the manuscript to address the reviewers’ comments using track changes and outlined the responses below. Thank you for giving us the opportunity to revise and resubmit the manuscript. I appreciate your time and look forward to your response.

Kind Regards,

Geoff

Dr Geoff McCombe

School of Medicine,

University College Dublin,

Dublin,

Ireland.

[geoff.mccombe@ucd.ie](mailto:geoff.mccombe@ucd.ie)

Journal Requirements:

1. Please amend your detailed Financial Disclosure statement. This is published with the article. It must therefore be completed in full sentences and contain the exact wording you wish to be published.

a. State the initials, alongside each funding source, of each author to receive each grant.
b. State what role the funders took in the study. If the funders had no role in your study, please state: “The funders had no role in study design, data collection and analysis, decision to publish, or preparation of the manuscript.”
c. If any authors received a salary from any of your funders, please state which authors and which funders.

Response

The following text has been added in the acknowledgements section of the manuscript and also in the Financial Disclosure statement:

We are grateful to the Ireland East Hospital Group, UCD College of Health & Agricultural Sciences and UCD School of Medicine who funded the salaries of authors GM and JB for the duration of their work on the study. The funders had no role in study design, data collection and analysis, decision to publish, or preparation of the manuscript.

Please review your reference list to ensure that it is complete and correct. If you have cited papers that have been retracted, please include the rationale for doing so in the manuscript text, or remove these references and replace them with relevant current references. Any changes to the reference list should be mentioned in the rebuttal letter that accompanies your revised manuscript. If you need to cite a retracted article, indicate the article's retracted status in the References list and also include a citation and full reference for the retraction notice.

Response

Reference list has been reviewed. There were no references added or removed from original submission.

Additional Editor Comments (if provided):


[Note: HTML markup is below. Please do not edit.]

Reviewers' comments:

Reviewer's Responses to Questions

<font color="black"><b>Comments to the Author</b><br><br> 1. Does this manuscript meet PLOS Digital Health’s <a href="<https://journals.plos.org/digitalhealth/s/journal-information#loc-criteria-for-publication>" target="_blank">publication criteria</a>? Is the manuscript technically sound, and do the data support the conclusions? The manuscript must describe methodologically and ethically rigorous research with conclusions that are appropriately drawn based on the data presented.</font>


Reviewer #1: Yes

Reviewer #2: Yes

Reviewer #3: Partly

Response

We have revised the manuscript based on the recommendations offered by the reviewers as well as provided answers to reviewers’ questions and hope that it has significantly improved the quality of the manuscript and now fully meets the PLOS Digital Health’s publication criteria.

--------------------

<font color="black">2. Has the statistical analysis been performed appropriately and rigorously?</font>

Reviewer #1: N/A

Reviewer #2: Yes

Reviewer #3: N/A

--------------------

<font color = "black">3. Have the authors made all data underlying the findings in their manuscript fully available (please refer to the Data Availability Statement at the start of the manuscript PDF file)?<br><br> The <a href="<https://journals.plos.org/digitalhealth/s/data-availability>" target="_blank">PLOS Data policy</a> requires authors to make all data underlying the findings described in their manuscript fully available without restriction, with rare exception. The data should be provided as part of the manuscript or its supporting information, or deposited to a public repository. For example, in addition to summary statistics, the data points behind means, medians and variance measures should be available. If there are restrictions on publicly sharing data—e.g. participant privacy or use of data from a third party—those must be specified.</font>

Reviewer #1: Yes

Reviewer #2: Yes

Reviewer #3: No

Response

This study is a scoping review of the literature on our study topic. As such, data for this study is the contents of the published articles included in this review. The list of articles reviewed in this manuscript can be accessed via Table 2 and the reference list. Table 2 also outlines specific details of the data that was extracted from the included articles for the purpose of this study.

--------------------

<font color = "black">4. Is the manuscript presented in an intelligible fashion and written in standard English?<br/><br/>PLOS Digital Health does not copyedit accepted manuscripts, so the language in submitted articles must be clear, correct, and unambiguous. Any typographical or grammatical errors should be corrected at revision, so please note any specific errors here.</font>

Reviewer #1: Yes

Reviewer #2: Yes

Reviewer #3: Yes

--------------------

<font color = "black">5. Review Comments to the Author<br/><br/>Please use the space provided to explain your answers to the questions above. You may also include additional comments for the author, including concerns about dual publication, research ethics, or publication ethics. (Please upload your review as an attachment if it exceeds 20,000 characters)</font>

Reviewer #1: The authors are commended for addressing an important research question and summarising the findings of the evidence base. The paper is well written and structured, concise, and of adequate English language standard. The paper has merit, but would benefit from some minor revisions. The following comments are intended to improve the paper:

Response

Thank you for your feedback.

1. PRISMA has been updated in a 2020 Statement. Inclusion of the updated PRISMA flowchart could be considered for your paper. This will allow elaboration of minor search details that are lacking in the body of the text, including: specific reasons for article exclusion, number of full texts unable to be retrieved, number of grey literature vs hand selected articles.

Response

Thank you for your recommendation. We have now replaced our PRISMA flow-chart with the PRISMA 2020 flow diagram for new systematic reviews which included searches of databases, registers and other sources (Figure 2: PRISMA Flow diagram).

2. Could you elaborate on the outcomes from the consultation with experts in the field and how this informed the inclusion of articles? i.e. who was consulted? what was this process? how many studies were included/excluded? Could you also clarify the purpose of ‘Step 6: Consultation’: was this to determine the articles’ quality or content related to the field of telemedicine?

Response

The following text has been added to Stage 6 of the methodology:

We consulted with healthcare professionals within the Irish healthcare system (GPs, infectious disease consultants) who had expertise in the treatment of COVID-19 and experience of using telemedicine consultations during the pandemic. The experts were recruited from the professional networks of the research team via purposive sampling. The consultation process resulted in no further studies being included or excluded from the final review. However the experts did provide advice as to the direction of future empirical inquiry in the topic area.

3. Acknowledgement of not undertaking quality assessment of included studies in a scoping review is noted in the discussion section under methodological concern. The paper may be strengthened by explicitly cautioning the reader of interpretation of the findings based on the absence of a quality assessment of included studies.

Response

We have now updated the section where we acknowledged not undertaking quality assessment by adding the below sentence.

*We therefore advise readers to take that into consideration when interpreting our findings.*

4. The finding that some aspects of telemedicine in GP practice were perceived by GPs as equivalent to face-to-face consultations is of interest. Were there any findings from studies on patient perspectives regarding telemedicine vs face-to-face?

Response

The following text was added to ‘Patient Satisfaction’ theme in Results section.

*However some studies highlighted patients’ preference for face-to-face consultations and their resistance to change (30, 31).*

5. Patient satisfaction with telemedicine was high. Were there additional factors to costs affecting patient satisfaction of telemedicine?

Response

The following text was added to the sentence about cost:

*……. while some patients also viewed technology-related knowledge as a barrier to satisfaction (23).*

6. Certain patient groups (young, urban, educated, working) were identified as being suitable to telemedicine. This may lead into the discussion about future research into barriers to using and accessing technologies for disadvantaged patient groups that would benefit from telemedicine.

Response

We thank the reviewer for their insight. We have now included a sentence in the Discussion section recommending future studies to further investigate barriers for receiving telemedicine care in GP practices when targeting disadvantaged patient populations.

*There were several patient-groups who reported better engagement with telemedicine; these were patients who were younger, coming from an urban setting, were highly educated and employed. Previous research has identified various factors that related to the likelihood of telemedicine utilization, which include patient demographic factors (e.g., age, race/ethnicity, sex, educational level), technological factors (e.g., available smartphone or another digital device, available broadband connection), patient and clinical factors (e.g., digital literacy, trust in electronic communication and care) and health system factors (e.g., appropriate data collection to ensure quality improvements) [1]. Future studies should investigate the ways on how to overcome these barriers when telehealth services are delivered by GP practices.*

7. As the world moves on from the COVID-19 pandemic, this paper will be an important reference on the immediate uptake on telemedicine in GP practice during the pandemic. Could you comment on what this paper’s findings/ themes say about the sustainability of telemedicine in clinical practice. What factors are important for telemedicine’s ongoing use, such that clinical practice does not revert back to pre-pandemic ways of working?

Response

While we appreciate the reviewer’s comment, we feel we have adequately discussed this in the ‘Implication for Research and Practice’ and ‘Conclusions’ sections of the discussion.

Reviewer #2: Thank you for this manuscript.

Please check all your references if they have the correct numbering in the text. For example, on page 6 of the paper, line 121-124 you mention the six-stage framework by Arksey & O' Malley and you cite a paper by Alan MMD et al which is not relevant.  I can see that you have the reference from Arksey & O' Malley on number [36] but this is not cited appropriately.

Response

Thank you for your feedback. We have revised our references.

--

Reviewer #3: General Comments

=================

Thank you for the privilege to review this manuscript, titled “Use of telemedicine in general practice in Europe since the COVID-19 pandemic: a scoping review of patient and practitioner perspectives. 
The aim of this study was “to conduct a scoping review of the literature examining the use of telemedicine for delivering routine general practice care since the start of the pandemic from the perspectives of patients and practitioners”. Indeed, this is a timely and relevant piece of work, and the authors are commended for that effort.

Response

We thank you for your feedback.

The authors used the six-stage framework developed by Arksey and O’Malley, blended with recommendations by Levac et al to review the existing literature on the subject matter. However, further works are needed on the explanation of the method used for this study. A brief description of the Arksey and O’ Malley framework will provide the reader with a better introduction to the framework, rather than leaving it to the reader to go and find out the paper discussing this framework.

Response

We thank you for your suggestion. We have revised the manuscript accordingly, by including a brief description of the Arksey and O’Malley framework.

*The process includes the following six steps: (1) identify the research question, (2) identify relevant studies from selected databases with a predefined terminology that will be used during the search, (3) select studies by filtering out the articles from the initial search that are not relevant to our research question, (4) chart the data using the narrative-review approach, (5) collect, summarise and report key findings, and (6) consult with experts in the area being studied (19). Based on this scoping review process, current research gaps in the area being studied were also addressed.”*

Specific Comments
=================
This paper would need some revisions to allow for clarity and consistence to the reader. The details of which are indicated in the subsequent comments in this report. 

Major Comments

-----------------
Abstract
1.      The abstract is fairly presented. However, in line 25 the word to be used is “adapt” instead of “adopt”.

Response

We have fixed this spelling error.

2.      In line 36, the correct word to use is “analyse” instead of “interpret” as the stated method is not used to interpret but to thematically analyse the data.

Response

We have changed the wording accordingly.

3.      In line 79, how are the authors defining a “bigger country”? They need to qualify this phrase.

Response

This phrase has been edited to read *‘Countries with large populations living in remote areas have higher usage of telemedicine (6).’*

4.      In line 85, please use “At that time” instead of “At this time”  and also replace “The” with “the”

Response

We have changed the wording accordingly.

Background
1.      In line 61, add a coma after the word pandemic.

Response

We have added a coma after the word “pandemic”.

Methodology
1.      Authors should state their justification of using a scoping review method instead of other available options.

Response

The chosen methodology was a scoping review which offers several advantages over other available options including the ability to examine exploratory research questions and determine gaps in the literature and identify areas for future empirical work. Furthermore, a scoping review also provides an opportunity for consultation with experts in the field to confirm the studies included in the review provide a comprehensive mapping of the literature on the topic.

2.      A brief description of the Arksey and O’ Malley framework will provide the reader with a better introduction of the framework, rather than leaving it to the reader to refer to the paper discussing this framework, before understanding this manuscript.

Response

We have revised the manuscript accordingly, by including a brief description of the Arksey and O’Malley framework.

*The process includes the following six steps: (1) identify the research question, (2) identify relevant studies from selected databases with a predefined terminology that will be used during the search, (3) select studies by filtering out the articles from the initial search that are not relevant to our research question, (4) chart the data using the narrative-review approach, (5) collect, summarise and report key findings, and (6) consult with experts in the area being studied (19). Based on this scoping review process, current research gaps in the area being studied were also addressed.*

3.      The search string strategy for the chosen database is better presented in a Table form rather than in a Figure form.

Response

We have now presented the search string strategy in a table format.

4.      In the paragraph beginning at line 201, I recommend that authors list all the countries involved in the study, even though they are later shown in the data charting stage.

Response

We have now typed out all the countries involved in the study in the main body of text.

*The included studies were from nine different countries (UK, Belgium, Denmark, Germany, Romania, Norway, Italy, Spain and France).*

5.      Why is the C (Critical) capitalized in line 206?

Response

There is no specific reason why “C” in the word “critical” was capitalized – we have now changed it to a lowercase “c”.

6.      These are referred to as open ended questions. Please correct all entries accordingly.

Response

We have corrected all entries accordingly, changing the word “style” to “ended”.

7.      In line 187, who are these experts? A clear definition is required as well as the selection criteria used to enrol these experts.

Response

The following text has been added to Stage 6 of the methodology:

*We consulted with healthcare professionals within the Irish healthcare system (GPs, infectious disease consultants) who had expertise in the treatment of COVID-19 and experience of using telemedicine consultations during the pandemic. The experts were recruited from the professional networks of the research team via purposive sampling.*

Results
1.      In line 211, add “s” at the end of the word “practitioner”

Response

In this sentence we meant to say that out of thirteen studies that focused on practitioners (practitioner-related studies), six addressed the issue of GP’s experience of telemedicine. Hence, we have now specified this by changing the sentence as follows:

*Six of the 13 practitioner-related studies addressed the issue of GPs’ experience of telemedicine, and their knowledge of the issue.*

Discussions
1.      Under methodological challenges, please discuss also the expert sampling biases.

Response

The following text has been added:

*We realise the recruitment of experts from the professional networks of the research team via purposive sampling will have led to an element of selection bias. However, the aim was to focus on a small sample of experts in the topic area to confirm the studies included in the review provide a comprehensive mapping of the literature on the topic and to provide advice as to the direction of future empirical inquiry in the topic area.*

2.      Comparison with existing literature, in line 317 to 318, authors subscribe to a certain belief. Could you please explain the basis of that statement, by providing undisputed evidence?

Response

In the following sentence we meant to say that the authors who wrote the article (Doraiswamy et al, 2020) believe that many aspects of telemedicine will remain after the Covid-19 pandemic. In this article, which is also a scoping review, the authors point out to a sentiment expressed by majority of healthcare professionals that they are eager to continue research on telehealth in the future. We have clarified this, by rewriting the below sentence.

*Based on their review, the authors observed a sentiment, expressed by majority of healthcare professionals, that they are eager to continue advancing telemedicine research and healthcare services in the future, post COVID-19 pandemic (36).*

Reference
I am afraid, that the authors seem to lack attention to detail in matters of referencing, yet there are clear guidelines with examples for building up Plos One references. Almost 25% of the references have errors. Please follow the specific guidelines instructions and correct the identified errors accordingly.  
Response

Reference errors corrected.

Ethics Statement
There is an omission of the study statement of ethics, indicated that the cohorts consented to the study.

Response

Since our article is a scoping review, it does not require a mention of cohorts consenting to the study since we use publicly available data that has already been published in peer-reviewed journals.

Other Comments
1.      Data Availability Statement, Authors provided the data availability statement that in not in line with Plos One requirements. They did not specify where the minimal data set underlying the results described in their manuscript can be found. This data should be available in Plos One recommended places such as public repository sites or provided as a supplementary file.
As a reviewer of this manuscript, I should be able to easily access this data set for verification of certain issues. Can the authors therefore, provide this minimal dataset?

Response

This study is a scoping review of the literature on our study topic. As such, data for this study is the contents of the published articles included in this review. The list of articles reviewed in this manuscript can be accessed via Table 2 and the reference list. Table 2 also outlines specific details of the data that was extracted from the included articles for the purpose of this study.

2.      Inconsistent placement of the full-stop (period) after the in-text citation number or before the in-text citation number.  For example (1). Or For example. (1)  I recommend you place the period after the citation number.

Response

We have corrected this inconsistency and have now placed all punctuation marks after the citation number.

--------------------

<font color = "black">6. PLOS authors have the option to publish the peer review history of their article (<a href="<https://journals.plos.org/digitalhealth/s/editorial-and-peer-review-process#loc-peer-review-history>" target="_blank">what does this mean?</a>). If published, this will include your full peer review and any attached files.<br><br>

<b>Do you want your identity to be public for this peer review?</b> If you choose “no”, your identity will remain anonymous but your review may still be made public.<br><br>

For information about this choice, including consent withdrawal, please see our <a href="<https://www.plos.org/privacy-policy>" target="_blank">Privacy Policy</a>.</font>


Reviewer #1: No

Reviewer #2: No

Reviewer #3: Yes: Benson Ncube

--------------------


[NOTE: If reviewer comments were submitted as an attachment file, they will be attached to this email and accessible via the submission site. Please log into your account, locate the manuscript record, and check for the action link "View Attachments". If this link does not appear, there are no attachment files.]

While revising your submission, please upload your figure files to the Preflight Analysis and Conversion Engine (PACE) digital diagnostic tool, <https://pacev2.apexcovantage.com/>. PACE helps ensure that figures meet PLOS requirements. To use PACE, you must first register as a user. Registration is free. Then, login and navigate to the UPLOAD tab, where you will find detailed instructions on how to use the tool. If you encounter any issues or have any questions when using PACE, please email PLOS at [figures@plos.org](mailto:figures@plos.org). Please note that Supporting Information files do not need this step.

__________________________________________________
In compliance with data protection regulations, you may request that we remove your personal registration details at any time.  (Use the following URL: <https://www.editorialmanager.com/pdig/login.asp?a=r>). Please contact the publication office if you have any questions.

1.	Lopez, A.M., K. Lam, and R. Thota, *Barriers and Facilitators to Telemedicine: Can You Hear Me Now?* American Society of Clinical Oncology Educational Book, 2021(41): p. 25-36.
